# Assessment of the Tolerance of a Chlorophyte *Desmodesmus* to CuO-NP for Evaluation of the Nanopollution Bioremediation Potential of This Microalga

**DOI:** 10.3390/nano13040737

**Published:** 2023-02-15

**Authors:** Svetlana P. Chebotaryova, Olga V. Zakharova, Alexander A. Gusev, Petr A. Baranchikov, Evgenii A. Kolesnikov, Anastasia S. Yakusheva, Elena V. Skripnikova, Elena S. Lobakova, Jingliang Xu, Md. Asraful Alam, Alexei E. Solovchenko

**Affiliations:** 1 Institute for Environmental Science and Biotechnology, Derzhavin Tambov State University, 392020 Tambov, Russia; 2 Department of Functional Nanosystems and High-Temperature Materials, National University of Science and Technology «MISIS», 119991 Moscow, Russia; 3 Engineering Center, Plekhanov Russian University of Economics, 117997 Moscow, Russia; 4 Faculty of Biology, Lomonosov Moscow State University, 119234 Moscow, Russia; 5 School of Chemical Engineering, Zhengzhou University, Zhengzhou 450046, China

**Keywords:** CuO-NP, microalgae, photosynthesis, oxidative stress, pigment composition, viability, bioaccumulation, bioremediation

## Abstract

Broad application of CuO nanoparticles (CuO-NP) for industrial and household purposes leads to a continuous increase in their discharge to, and, hence, ever-increasing environmental hazards for aquatic ecosystems. Microalgae-based technologies hold promise for bioremediation of diverse hazardous micropollutants (HMP), including NP, from wastewater. In this study, we tested the ability of the green microalga *Desmodesmus* sp. to accumulate CuO-NP or their components. We also assessed the tolerance of this microalga to the environmentally relevant concentrations of CuO-NP. Using scanning electron microscopy, we demonstrated that the average size of CuO-NP was 50–100 nm, and their purity was confirmed with elemental composition analysis. Tests of the colloidal suspensions of CuO-NP showed that the hydrodynamic diameter of CuO-NP and their aggregates was below 100 nm. Flow cytometry analysis showed that CuO-NP at a concentration of 100 µg L^−1^ slightly inhibited the viability of microalgae cells and led to an increase in their oxidative stress. The assessment of the condition of photosystem II showed that CuO-NP exert a multifaceted effect on the photosynthetic apparatus of *Desmodesmus* sp., depending on the concentration of and the exposure to the CuO-NP. *Desmodesmus* sp. turned to be relatively tolerant to CuO-NP. In addition, the ICP-MS method revealed increased bioaccumulation of copper by microalgae cells in the experimental groups. The outcomes of this study indicate that the *Desmodesmus* sp. has a significant potential for bioremoval of the copper-based nanostructured HMP from an aquatic environment.

## 1. Introduction

Since nanoparticles (NP) are extensively applied for commercial, environmental, and healthcare benefits, they are currently produced at an industrial scale [1,2]. CuO nanoparticles (CuO-NP) have become widespread, and their global production is forecasted to reach 1600 tons/year by 2025 [3]. Due to their unique chemical and physical properties, these NP have found broad applications in various commercial formulations, including agrochemicals, paints, semiconductor compounds, sensors, catalysts, and antimicrobial compounds [4]. In the process of production, transportation, storage, consumption, and disposal of the above-listed products, the CuO-NP are increasingly discharged into the environment; following diverse pathways, these NP end up in aquatic ecosystems [5,6].

In the aquatic environment, NP cause various impacts on its inhabitants. Thus, CuO-NP were reported to increase mortality, delay hatching, and distort heartbeat and embryo formation in *Danio rerio* [7]. Another study [8] showed that exposure to CuO-NP reduces the viability of sea urchin spermatozoa, disrupts mitochondrial activity, and increases the production of reactive oxygen species (ROS) and lipid peroxidation. CuO-NP inhibited growth, decreased carotenoid content, and deteriorated cellular metabolism of the microalga *Chlamydomonas reinhardtii* [9] and the cyanobacterium *Lyngbya* sp. [10].

CuO-NP are known to induce oxidative stress and to exert cytotoxicity, genotoxicity, and immunotoxicity effects [11]. The release of metal ions from NP is among the main causes of the NP toxicity for many organisms [12]. At the same time, it is necessary to take into account the whole set of the NP toxicity determinants, including their physico-chemical parameters and concentration [11].

It is also known that accumulating NP in living organisms can be transferred between trophic levels in food chains with concomitant build-up of their concentration in the organisms toward the higher trophic levels [13]. The upward transfer of NP across trophic levels was studied in a two-level system consisting of *Artemia salina* and *Amatitlania nigrofasciata* [14]. It was reported that CuO-NP accumulated by *Chlorella vulgaris* was detected in the *Daphnia magna* they grazed [15]. Therefore, the upward transfer of NP across trophic levels poses a great danger, both for natural ecosystems and for humans.

The deteriorative effects of the industrial CuO-NP on the environment call for the development of knowledge-based approaches to mitigation of these effects. Accordingly, the development of methods for bioremoval of NP from the environment gains increasing attention. Since mechanical cleaning systems are energy-intensive, the potential of microbial biotechnologies for the bioremoval of micropollutants comes to the foreground. Thus, a promising solution to the problem of wastewater treatment to remove CuO-NP is constituted by using microalgae, the organisms with a long story of application in the field of bioremediation [16]. The resulting biomass of microalgae can be utilized for the production of biofuels [17,18] or other industrially important compounds [19].

Indeed, microalgae are capable of accumulation of CuO-NP [9,15,20]. Still, high concentrations of these NP are toxic to many microalgal species [9,10].

Therefore, the foundation of the biotechnology for bioremediation of CuO-NP containing wastewater using microalgae requires the selection of species that would be (i) resilient to environmentally relevant concentrations of the NP (up to 1 mg L^−1^) and (ii) capable of bioaccumulating the NP or their components. Therefore, the aim of the present study was to assess the CuO-NP resilience and the bioaccumulation potential of the microalga *Desmodesmus* sp. IPPAS S-2014 [21,22], which showed the greatest (compared with the microalgae from the genera *Lobosphaera* sp. IPPAS C-2047, *Micractinium* IPPAS C-2056, and other representatives of the genus *Desmodesmus*) retention of photosynthetic capacity in a preliminary screening of CuO-NP resilience (for more information, see Figure S1 and [23]).

## 2. Materials and Methods

### 2.1. Nanoparticles

#### 2.1.1. Nanoparticle Characterization

The CuO-NP from Sigma-Aldrich (St. Louis, MO, USA) were used in the work. The morphology and particle size were determined before the experiment by scanning electron microscopy (SEM) on a Tescan Vega 3 microscope (Czech Republic). The elemental analysis was carried out by the method of energy dispersive X-ray spectroscopy (EDX) using the EDX analyzer “10 mm^2^ SDD Detector-X-Act” (Oxford Instruments, Oxford, UK).

#### 2.1.2. Obtaining of the NP Suspension

To introduce the NP into the algae cultivation medium, aqueous dispersions of the NP were prepared. The NP powder samples (0.5 mg) were weighed using ViBRA HT analytical scales (Shinko Denshi, Tokyo, Japan), placed into cylindrical tubes with screw caps with 10 mL of sterile distilled water (pH 7.1 ± 0.2). The suspensions were mixed and sonicated (VBS-41H, Vilitek, Moscow, Russia) for 10 min (the ultrasonic power—180 W, the bath volume—4 L) to obtain a 500 µg 10 mL^−1^ NP stock. The stock suspension was diluted to obtain the working suspensions of 50 µg 10 mL^−1^ and 5 µg 10 mL^−1^. To assess the stability of the obtained dispersed systems, the zeta potential of the NP in the suspensions was analyzed using the ZetasizerNanoZS analyzer (Malvern Panalytical, Malvern, UK) [24,25]. The size and the aggregates of the particles in the obtained colloidal solutions were determined by the method of dynamic light scattering (on the same ZetasizerNanoZS device) [24,26].

### 2.2. Testing of the Effect of the NP on Microalgae

A stress-resilient unicellular green alga *Desmodesmus* sp., strain IPPAS S-2014 (referred to herein as *Desmodesmus* sp.) was selected as the object of the study. This strain of microalgae was previously isolated from fragments of the invertebrate animal hydroid *Dynamena pumila* [21], but its tolerance to nanopollution has never been tested before.

The *Desmodesmus* sp. cells were cultivated in BG-11 medium [27] in glass conical flasks with a capacity of 250 mL containing 150 mL of the culture under constant illumination with white LED lamps (480 µM PAR quanta/m^2^/s) at a temperature of 27 °C and pH 7.0–7.2.

#### 2.2.1. Experimental Design

The experiment was started by the addition of NP suspension to the test culture of the microalga. Specifically, 6-mL aliquots of the microalgal culture (OD = 0.6 at 560 nm) were introduced into each of well of the 6-well plate (Eppendorf, Hamburg, Germany) together with either 120 µL of the corresponding NP suspension or 120 µL of distilled water (the control) and gently mixed. The final NP concentration was 10, 100, or 1000 µg mL^−1^, which is in the range of environmentally relevant NP concentrations [28,29,30].

#### 2.2.2. Cell Viability Tests

The changes in the viability of the NP-treated culture (expressed as % of that in the untreated control culture, see Figure S2) was considered the primary indicator of the microalgal culture condition and, correspondingly, the effect of the NP. This cell viability was assessed using the cell analyzer Muse Cell Analyzer, (Merck Millipore, Darmstadt, Germany) using the reagent Muse Count & Viability from the same manufacturer. Since the working cell suspensions were of high density, the sampled suspension was diluted (1:20) with fresh BG-11. After that, the cell cultures were further diluted with the Muse Count & Viability reagent (1:20) and incubated for 5 min before the measurement. The cell density of the culture samples was additionally determined in the NP-treated and the untreated control [31].

#### 2.2.3. Oxidative Stress Assessment

The degree of oxidative stress in the algal culture samples was gauged using the Muse Cell Analyzer and Muse Oxidative Stress reagent kit from the same manufacturer. The stock solution of the Muse Oxidative Stress reagent was diluted (1:100) with the 1× Assay buffer from the kit. The working solution was prepared by further diluting the stock solution with the 1× Assay buffer. The algal culture samples were also diluted (1:20) with the 1× Assay buffer. After that, 10 µL of the diluted cell suspension was added to 190 µL of the working solution. The obtained samples were incubated for 30 min at 37 °C before performing the measurements [32].

#### 2.2.4. Photosynthetic Pigment Assay

The pigments in the microalgal samples were quantified spectrophotometrically [33] in dimethyl sulfoxide (DMSO) extracts. Briefly, 1 mL of the cell suspension was centrifuged (5 min, 5000× *g*) on a MiniSpin centrifuge (Eppendorf, Hamburg, Germany). The supernatant was discarded, and the cell pellet was incubated in DMSO at 70 °C for 10 min with intensive stirring and the cells’ debris was then removed by centrifugation.

The concentrations of chlorophylls *a* and *b*, as well as the total carotenoids in the extract was carried out spectrophotometrically in 96-well plates on a Multiskan Sky spectrophotometer (Thermo Scientific, Waltham, MA, USA) using the equations reported in [33]:

C_Chl *a*_ (mg/L) = 13.44 D_666_ − 4.85 D_650_; (1)

C_Chl *b*_ (mg/L) = 24.58 D_650_ − 6.65 D_666_; (2)

C_Car_(mg/L) = (1000 D_480_ − 1.29 C_Chl *a*_ − 53.76 C_Chl *b*_)/220 (3)

where C_Chl *a*_, C_Chl *b*_, and C_Car_ are the concentrations of chlorophylls *a* and *b* and carotenoids, respectively; and D*_λ_* is the optical density of the extract at the wavelength *λ* (nm).

#### 2.2.5. Measurement of Photosynthetic Activity of the Microalgae

The photosynthetic activity of the microalgae was assessed as a potential maximal photochemical quantum yield of photosystem 2, PS II Q_y_ = (Fm − Fo)/Fm = Fo)/Fm, where Fo is the minimum and Fm is the maximum intensity of Chl, a fluorescence excited with a weak measuring light and a strong saturating pulse, respectively, in the microalgal cells dark-adapted for 15 min. The PS II Q*_y_* was measured with a PAM-fluorometer FluorPen FP S100 (Photon Systems Instruments, Drasov, Czech Republic) according to the manufacturer’s protocol. The PS II Q_y_ parameter is frequently used to monitor direct and PSII-mediated stress effects on photosynthetic organisms. Generally, any damage to the cells directly or indirectly causing inhibition of the photosystem II or damage to its reaction centers leads to an observable decline on Fv/Fm [34].

### 2.3. Assessment of the NP Absorption and/or Internalization by the Microalgal Cells

The content of the NP or their components in the microalgal cells and the residual NP content of particles in the medium were determined by inductively coupled plasma mass spectrometry (ICP-MS) on an ICP-MS spectrometer (Bruker, San Jose, CA, USA). The samples for the analysis were prepared by filtering 2 mL of cell suspension through a membrane filter (Millipore, Burlington, MA, USA) with a pore size of 0.45 microns. The microalgal culture incubated with the maximum concentration (1000 µg mL^−1^) of the NP was used for this analysis. Since large aggregates of NP could be detained by the filter in addition to the NP accumulated by/adsorbed on the microalgal cells, a reference analysis was conducted by filtering the nutrient medium containing the same amount of the NP, but the lacking microalgal cells culture was additionally filtered. The obtained data were taken into account when calculating the NP bioaccumulation efficiency. Furthermore, to avoid an error in the determination of the Cu content due to handling the cells deposited on the filter, we re-measured the cell density of the cell suspension washed from the filter (it was actually used for the ICP assay) and corrected the final result accordingly.

For the analysis, the biomass samples were mineralized with a mixture of saturated HCl and HNO_3_ (3:1, vol./vol.). The efficiency of the bioaccumulation/adsorption of the NP by the microalgae cells was calculated via the following equation [35,36]:

Bioaccumulation/adsorption (%) = (C*_i_*/C*_f_*)·100% (4)

where C*_i_* is the NP concentration in the microalgal cell sample and C*_f_* is the initial NP concentration in the medium.

### 2.4. Statistical Treatment of the Data

All the experiments were carried out in three biological repetitions with three technical samplings for each biological replica. The statistical significance of the difference of the main indicators of the culture condition (chlorophyll and carotenoid contents, cell density, cell viability, and the oxidative stress degree) was evaluated using the Student’s *t*-criterion (at the 0.05 significance level). For the Fv/Fm parameter, a nonparametric Mann–Whitney U-test was used (at the 0.05 significance level) since the chlorophyll fluorescence parameters generally do not feature the normal distribution [37]. The average values and their standard deviations are shown in the figures and tables. Statistical treatment of the data has been carried out with Excel spreadsheet software (Microsoft, Redmond, WA, USA).

## 3. Results

### 3.1. Characterization of the CuO-NP

**Figure 1:**
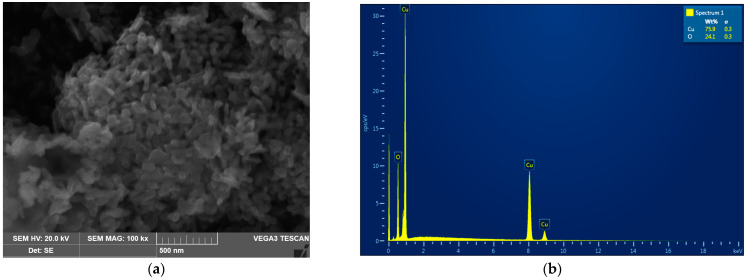
The analysis of the CuO-NP used in this work. (**a**) Scanning electron micrograph. (**b**) Element composition (EDX spectrum).

The analysis of the CuO-NP revealed that the NP used in this work were rod-shaped with rounded ends, assembled into aggregates (Figure 1a).

As can be seen from the presented microphotographs, the individual NP were, on average, 50 nm in diameter and 70–100 nm long. Energy dispersive X-ray spectroscopy showed that the analyzed NP powder is composed of copper oxide without any impurities (Figure 1b). Collectively, both analyses confirmed that the studied NP samples were indeed represented by nano-sized (50–100 nm) particles of pure CuO.

### 3.2. Properties of the Colloidal Systems of the NP

**Figure 2:**
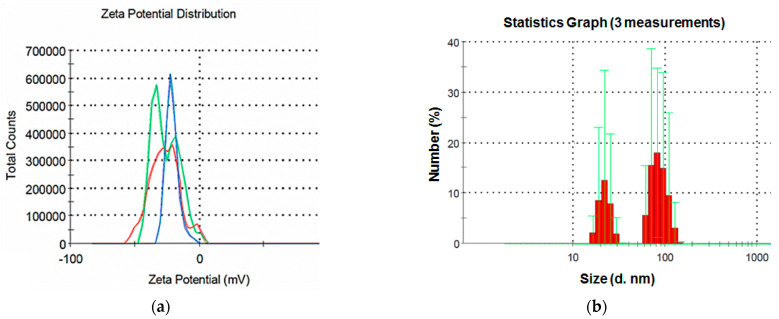
The CuO-NP suspension (**a**) zeta potential and (**b**) particle size distribution.

The studied NP suspension featured a zeta potential of 21.4 mV (Figure 2a), which indicates a relatively low stability of the dispersed system.

The results of the analysis of the CuO particle size in the colloidal solutions demonstrate a bimodal particle size distribution (Figure 2b). The first peak is in the range of 20–30 nm, the second is 70–100 nm. The results are consistent with the SEM data obtained in this study, and the presence of two peaks can be explained by the elongated shape of the particles, as well as their aggregation in the aqueous environment.

In general, the results of the analysis of the dispersed composition of colloidal suspensions of the NP show that, despite the use of sonication, the NP aggregated in the aqueous medium, but the size of the aggregates was not large.

### 3.3. CuO-NP Effects on the Microalgal Cultures

#### 3.3.1. Cell Viability

**Figure 3:**
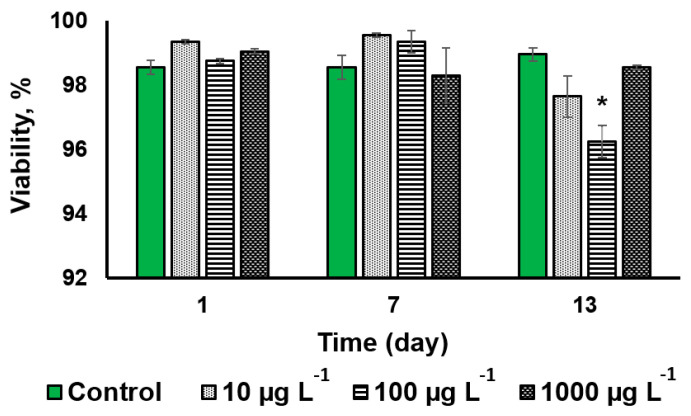
The analysis of the viability of *Desmodesmus* sp. cells incubated with CuO-NP as compared to the untreated cells (Values significantly differing from the control are marked with an asterisk, *).

The CuO-NP treatment at a concentration of 100 µg L^−1^ induced a statistically significant effect (2.7% decline of the cell viability) only on the 13th day of exposure (Figure 3 and Figure S2).

**Figure 4:**
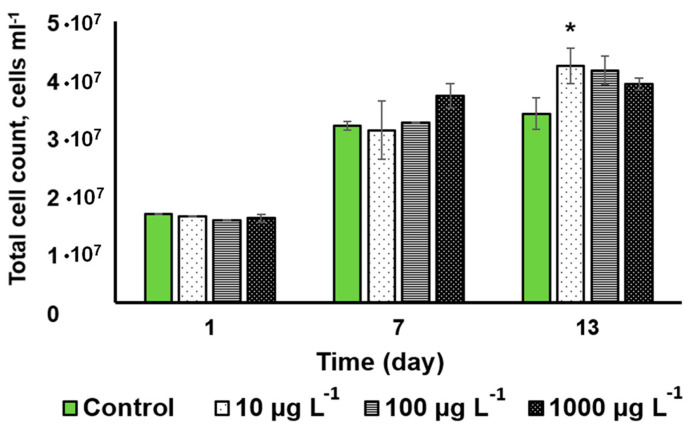
The changes in *Desmodesmus* sp. cell density in the cultures as a function of the added CuO-NP concentration (Values significantly differing from the control are marked with an asterisk, *).

Figure 4 shows the dynamics of the changes in the cell density of the cultures incubated with the CuO-NP and the untreated control. The CuO-NP exerted no significant effect on microalgal cell density except for a slight increase observed on the 13th day in the culture incubated with 10 µg L^−1^ CuO-NP.

Judging from the cell viability and cell density, the tested microalga *Desmodesmus* sp. turned out to be relatively resilient to the effects of CuO-NP.

#### 3.3.2. Oxidative Stress Severity

**Figure 5:**
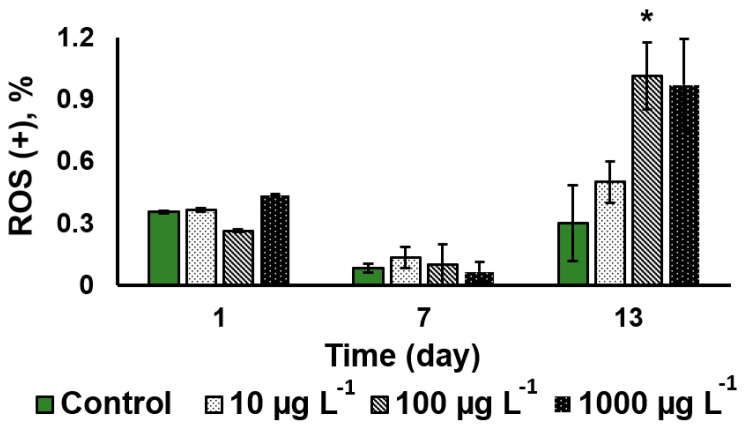
Comparative analysis of the oxidative stress severity in the *Desmodesmus* sp. cells as a function of the added CuO-NP concentration (Values significantly differing from the control are marked with an asterisk, *).

The assessment of the relative severity of oxidative stress to the algal cells showed that CuO-NP did not induce a sizeable an increase in the oxidative stress intensity during the first seven days of the NP exposure regardless of the CuO-NP concentration. It was only on the 13th day of the exposure to the 100 µg L^−1^ CuO-NP that a three-fold increase in the severity of oxidative stress was observed, as well as in the cultures treated with 1000 µg L^−1^ CuO-NP (Figure 5).

#### 3.3.3. Cell Pigment Composition

**Figure 6:**
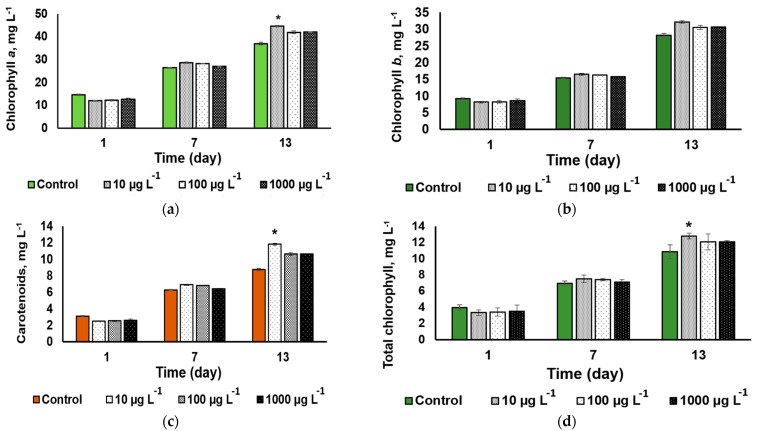
The dynamics of (**a**) chlorophyll *a*, (**b**) chlorophyll *b*, (**c**) total carotenoid, and (**d**) chlorophyll *a* + *b* contents in the cultures of *Desmodesmus* sp. incubated in the presence of different concentrations of CuO-NP (Values significantly differing from the control are marked with an asterisk, *).

A statistically significant CuO-NP effect on the microalgal cell pigment composition (a slight increase) was revealed only on 13th day of exposure to the NP concentration of 100 µg L^−1^ (Figure 6a,c). The same trend was revealed in the total chlorophylls (*a* + *b*) concentration of chlorophyll (Figure 6d). Otherwise, the cultures exhibited a steady accumulation of both pigment groups regardless of the added CuO-NP concentration, showing no sign of impairment of the pigment apparatus of the cell.

The calculation of per cell pigment content revealed no significant changes in the carotenid or chlorophyll content (Figure S3), therefore the increase in the volumetric pigment content in the variant “10 µg L^−1^” is caused by the increase in the cell number (Figure 4).

#### 3.3.4. Photosystem II Functional Condition

**Figure 7:**
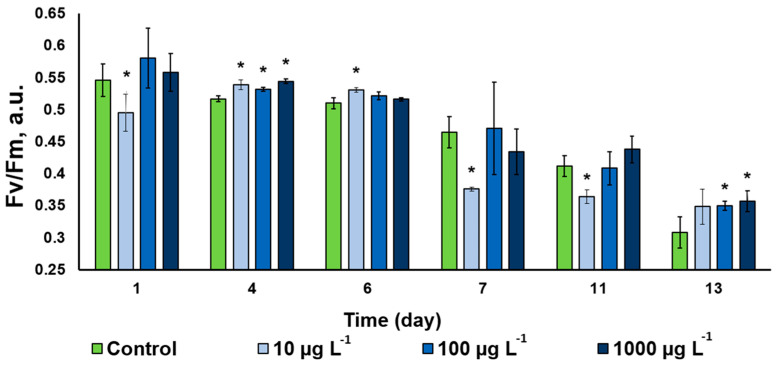
Changes of the Fv/Fm parameter in the of *Desmodesmus* sp. incubated with different CuO-NP concentrations (Values significantly differing from the control are marked with an asterisk, *).

The photochemical activity of the *Desmodesmus* sp. cells assessed as potential maximal quantum yield of photosystem II displayed a steady decline throughout the observation period (Figure 7). This trend was evident in the untreated control, likely due to slow-down of the cell division in the studied culture manifesting the onset of the stationary phase of the culture growth. The CuO-NP-treated cultures followed approximately the same trend. Interestingly, the NP in the lowest concentration used triggered a small decline of the PS II Qy, while the cultures treated with higher NP concentrations frequently possessed a higher photochemical activity than that in the control (Figure 7). There was no clearly expressed pattern in the CuO-NP effect on the functioning of the photosynthetic apparatus of the studied microalgal cultures. Intermittent inhibition and stimulation effects have been observed in our experiments on the background of the overall decline in the photosynthetic activity of the cultures taking place, regardless of the presence and/or concentration of the NP.

#### 3.3.5. Uptake and adsorption of the CuO-NP

The copper content assay showed that the volumetric amount of copper taken up and adsorbed by *Desmodesmus* sp. exposed to the 1000 µg mL^−1^ NP for 13 days was 330 µg L^−1^; the cell density of the culture increased ca. 2.5 times by that time (Figure 4). This amount is constituted by the copper adsorbed to the cell surface, as well as the copper internalized by the cells both as NP and in ionic form. These figures should be interpreted while taking into account the constitutive copper content integral to the enzyme molecules in the cell, as well as the copper taken up from the growth medium. The cell density of the culture in the beginning of the experiment was 1.36·10^7^ cells mL^–1^, which yield the “background” level of ca. 4.23 fg Cu per cell. The results of the copper assay in the different sample types including the filtrates of the medium are shown in Table 1.

Table 1 shows that the microalgal culture unexposed to CuO-NP contained 57.6 µg L^−1^ copper, whereas the CuO-NP-exposed culture Cu contained six times more copper. The efficiency of bioaccumulation and adsorption of CuO-NP (or copper contained therein) by *Desmodesmus* sp. cells was 33%, meaning that the concentration of CuO-NP in the growth medium was reduced by ca. 33%. Notably, the ICP-MS analyses cannot distinguish between the copper in the NP and that within the microalgal enzymes. There are also reports on the ability of microalgae to absorb trace elements, including Cu, from the environment [38,39]. Therefore, some of the copper discovered in the *Desmodesmus* sp. cells under our experiment conditions can stem from the micro-salts of the BG-11 nutrient medium, but contribution of the medium cannot be significant. Thus, there was no increase in the copper content of the control culture cells. Collectively, our findings suggest that the NP or their components were the major source of the Cu discovered in the NP-treated cells.

## 4. Discussion

Taken together, the results of the biochemical and functional tests suggest that the microalga selected as the test object for our study turned out to be quite resilient to the CuO-NP in the medium, even at concentrations as high as 1000 µg L^−1^. Moreover, under the conditions studied, the cells of the microalgae exhibited a pronounced ability to take up copper from the nutrient medium. The latter observation is very important in the context of the development of biotechnology for bioremediation on nanopolluted wastewater, as well as water with a high concentration of ionic copper forms.

At the same time, CuO-NP at a concentration of 100 µg L^−1^ were able to reduce the viability of the microalgae culture, likely via enhancing ROS production exacerbating the oxidative stress [40], which was also observed in these experimental variants.

The ‘nonlinear’ toxic effect of the studied NP is consistent with the results obtained in our previous work [41] and other studies [42,43]. Such a toxicity pattern may be associated with stepwise kinetics of the acclimation of the microalga to the NP-induced stress, the cross-talk of the NP-induced effects, and the cellular signaling [44]. Studies on the patterns of the slightly expressed toxicity are very important for understanding the momentary and accumulated effects of hazardous micropollutants, as well as for control of undesirable microflora development with chemicals acting in low doses.

Interestingly, all the cultures studied displayed a steady accumulation of the photosynthetic pigments, regardless of the presence of the CuO-NP or their concentration. Most likely, this finding reflects a steady increase in cell density [10], although direct counting of the cells could not be performed under our conditions due to the cell aggregate formation. An indirect indication on the culture growth and the onset of the stationary phase by the 13th day of cultivation is the decline in PS Qy taking place due to over-reduction of the electron transport chain components in the chloroplast membranes of the cell. Although many studies indicate that copper is deteriorative for the photosynthetic apparatus [45,46,47,48], in our study, the CuO-NP and the ionic forms of Cu potentially present in the medium did not exert a profound inhibitory effect of PS II. The intermittent decline in photosynthetic activity recorded may be associated with Cu^2+^ ions likely released from the NP surface during sonication of the NP suspensions [40]. Another plausible source of the ‘nonlinear’ pattern of the NP toxicity can be the diversity of the NP size and shape [43].

The specific mechanisms of CuO-NP- and Cu^2+^-mediated inhibition of photosynthesis are related to the disruption of the oxygen-evolving complex of photosystem II [49]. Copper ions also affect photosynthesis via interaction with the electron transport chain and interfering with the uptake of Fe ions essential for the biosynthesis of porphyrins, especially cytochromes [50]. Overall, the main mechanism of CuO-NP toxicity is the release of Cu ions [20,51,52]. In addition to this, CuO-NP and their aggregates are able to attach to the surface of microalgae cells, leading to direct or indirect toxicity through physical damage, internalization, or light shading [53]. We also found that the CuO-NP used in our experiment tended to aggregate in the aqueous medium, possibly causing some shading of the cells.

Metal NP are also able to interfere with the enzymes of the antioxidant system, triggering the processes mimicking the response of the plant’s low-molecular antioxidant system to stress [47,54]. It is possible that the increase in carotenoids in the microalgae cells, which was observed in our study, reflects an antioxidant response that was quite efficient, judging from the low percentage of the cells exhibiting the symptoms of oxidative stress.

*Desmodesmus* sp. was able to uptake and/or absorb CuO-NP or copper emitted from the NP from the aqueous medium. The exact pathways of NP uptake by the microalgae were not considered in this study, although it is possible that the internalization of NP proceeded via energy-dependent clathrin-mediated endocytosis, as reported in [53]. To be internalized by the algal cells, NP need to penetrate two barriers—the cell wall and the cytoplasmic membranes. In the case of microalgae, relatively thick and rigid cell walls are usually considered as the first and the toughest barrier preventing internalization of NP. At the same time, the cell walls of algae are semi-permeable and usually porous. The pore diameter is normally in the range of 5–20 nm, thereby allowing smaller NP to readily pass through the cell wall [55]. In our study, the average diameter of the individual CuO particles was about 50 nm, and their length was 70–100 nm, therefore, they would hardly be able to pass the cell wall for subsequent internalization by the microalgae cells. Accordingly, adsorption of NP on the cell surface but not uptake can be suggested as the main mechanism of the CuO-NP bioremoval from the medium. Still, NP can alter the cell wall and membrane properties, facilitating their penetration into the cells [50], therefore this issue requires further studies.

To conclude, the efficiency of bioremoval of the CuO-NP or the copper they harbor was around 33%, which makes the *Desmodesmus* sp. a potential vehicle for bioremediation of wastewater contaminated with Cu-containing NP. The efficiency reached in this work can be improved using physico-chemical and biotechnological approaches facilitating the adsorption of the NP on the surface of, as well as uptake of the NP by the cells. One of the ways of accomplishing this is the selection of microalgal strains for increasing their NP (ad)sorption capacity, isolating or constructing the microbial consortia with synergistically increased NP bioremoval efficiency. Another promising approach is the chemical and/or physical stimulation of the ability of microalgae to the biotransformation of NP [50] to better mitigate the negative consequences of nanopollution.

## Figures and Tables

**Table 1:** Copper content in different sample types.

Sample	Copper, µg L^−1^	Copper, fg/Cell
*Desmodesmus* sp. (the control)	57.6	4.23
*Desmodesmus* sp. after the CuO-NP exposure	367.2 *	9.18
The filtrate of BG-11 medium	632.8	-
The filtrate of the algal cell-free BG-11 medium with CuO-NP	962.8	-

* Including 37.2 µg L^−1^ of copper adsorbed on the filter (see Methods).

## Data Availability

The data are available from the corresponding author on reasonable request.

## Supplementary Materials

The following supporting information can be downloaded at: https://www.mdpi.com/article/10.3390/nano13040737/s1, Figure S1: Preliminary screening of CuO-NP effects on different microalgal strains via PS II Qy (Fv/Fm): (a) *Desmodesmus* sp. IPPAS C-2014; (b) *Lobosphaera* sp. IPPAS C-2047; (c) *Desmodesmus* sp. 1 Dp66E-1; (d) *Micractinium simplicissimum* IPPAS C-2053; Figure S2: Analysis of the viability of *Desmodesmus* sp. cells incubated with CuO-NP as compared to the untreated cells; Figure S3: The dynamics of (a) chlorophyll *a*, (b) chlorophyll *b*, (c) total carotenoid, and (d) chlorophyll *a* + *b* content per cells of *Desmodesmus* sp. Incubated in the presence of different concentrations of CuO-NP.
