# Supplementary material for: Assessment of the Tolerance of a Chlorophyte Desmodesmus to CuO-NP for Evaluation of the Nanopollution Bioremediation Potential of This Microalga
Source: Nanomaterials (Basel). 2023 Feb 15;13(4):737. doi: 10.3390/nano13040737 (PMC9959455; doi:10.3390/nano13040737)
Supplement: Supplementary file 1 [file nanomaterials-13-00737-s001.zip › nanomaterials-2156487-supplementary.pdf]

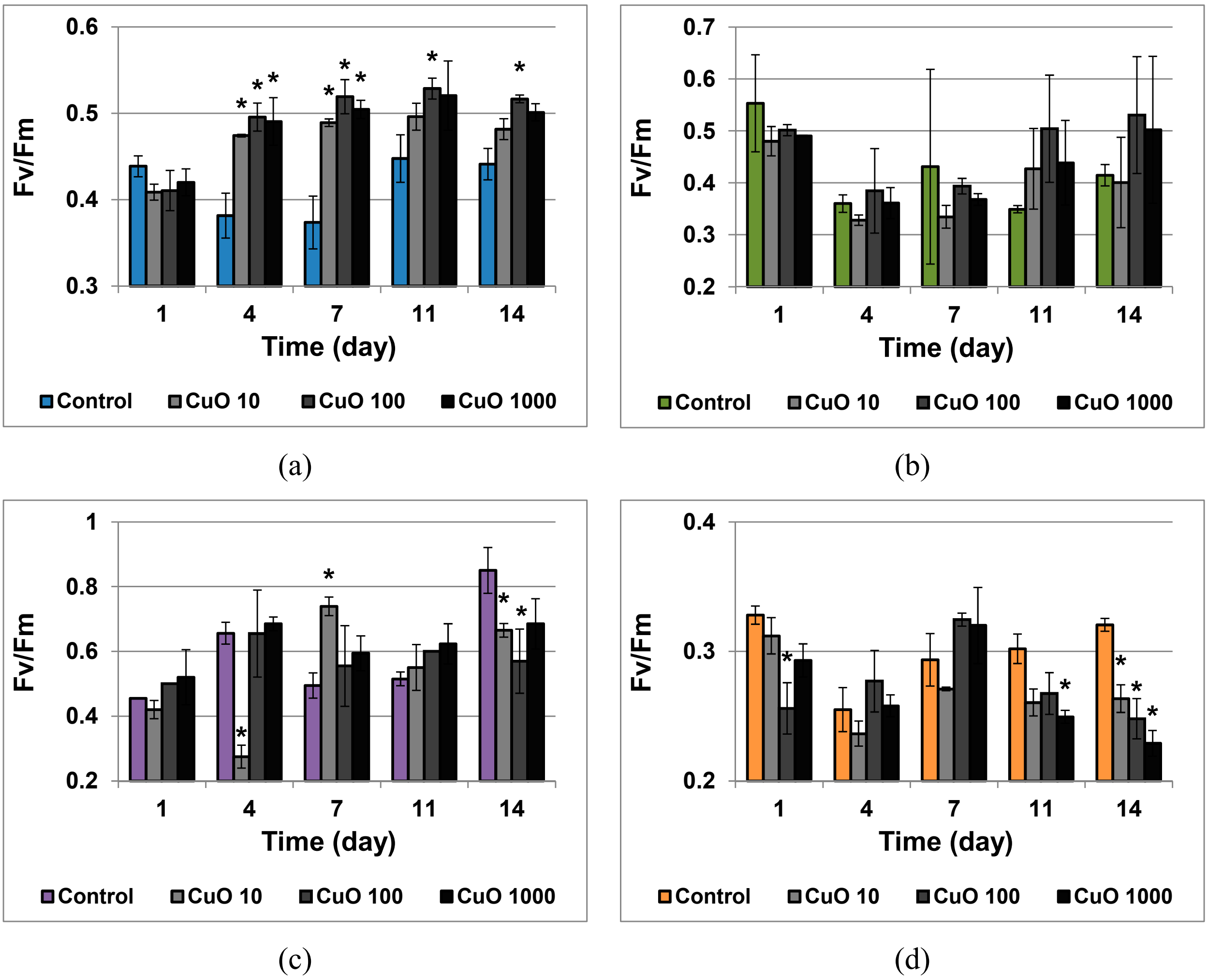

Figure S1. Preliminary screening of CuO-NP (10, 100 and 1000 μg L^{-1}) effects on different microalgal strains via PS II Qy (Fv/Fm): (a) *Desmodesmus* sp. IPPAS C-2014; (b) *Lobosphaera* sp. IPPAS C-2047; (c) *Desmodesmus* sp. 1 Dp66E-1; (d) *Micractinium simplicissimum* IPPAS C-2053. Average values significantly differing from the control at the 0.05 significance level are marked with asterisk, *.

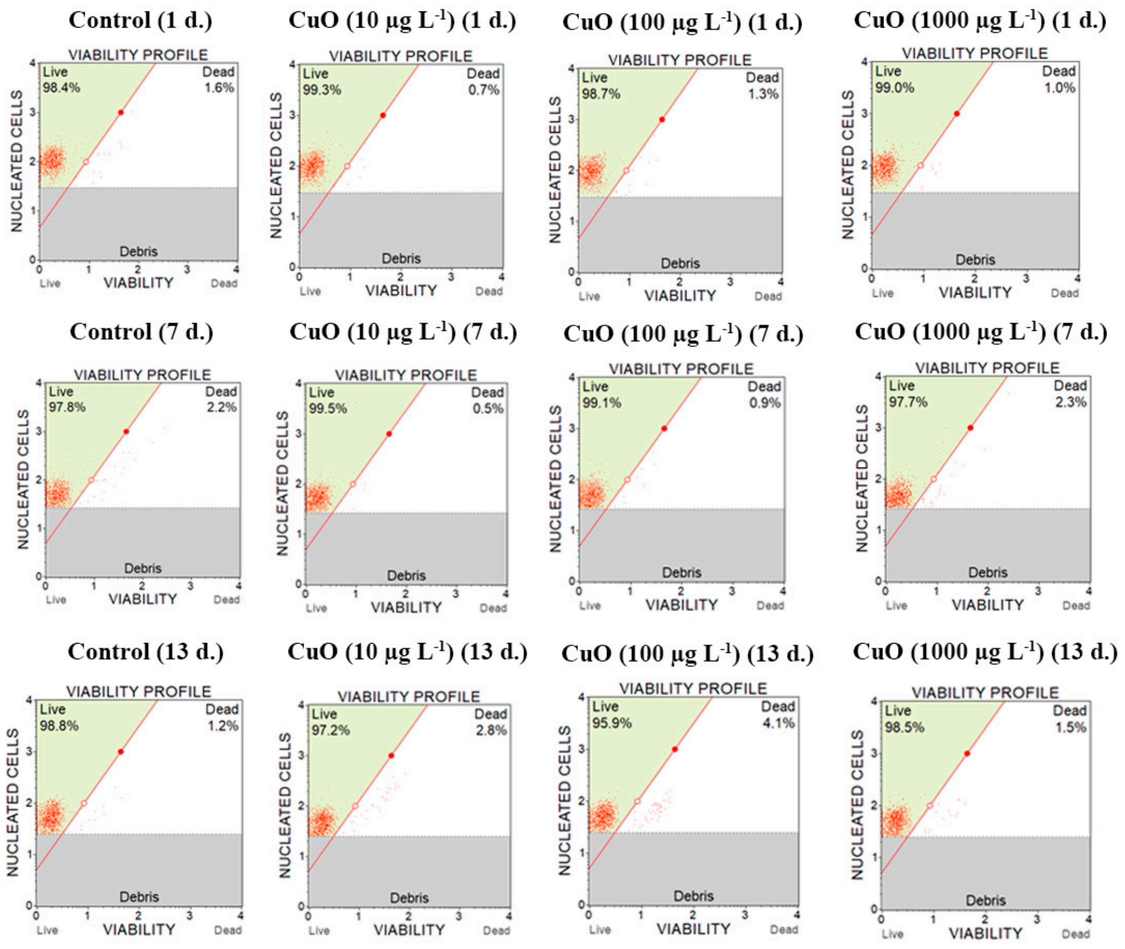

Figure S2. The analysis of the viability of *Desmodesmus* sp. cells incubated with CuO-NP as compared to the untreated cells.

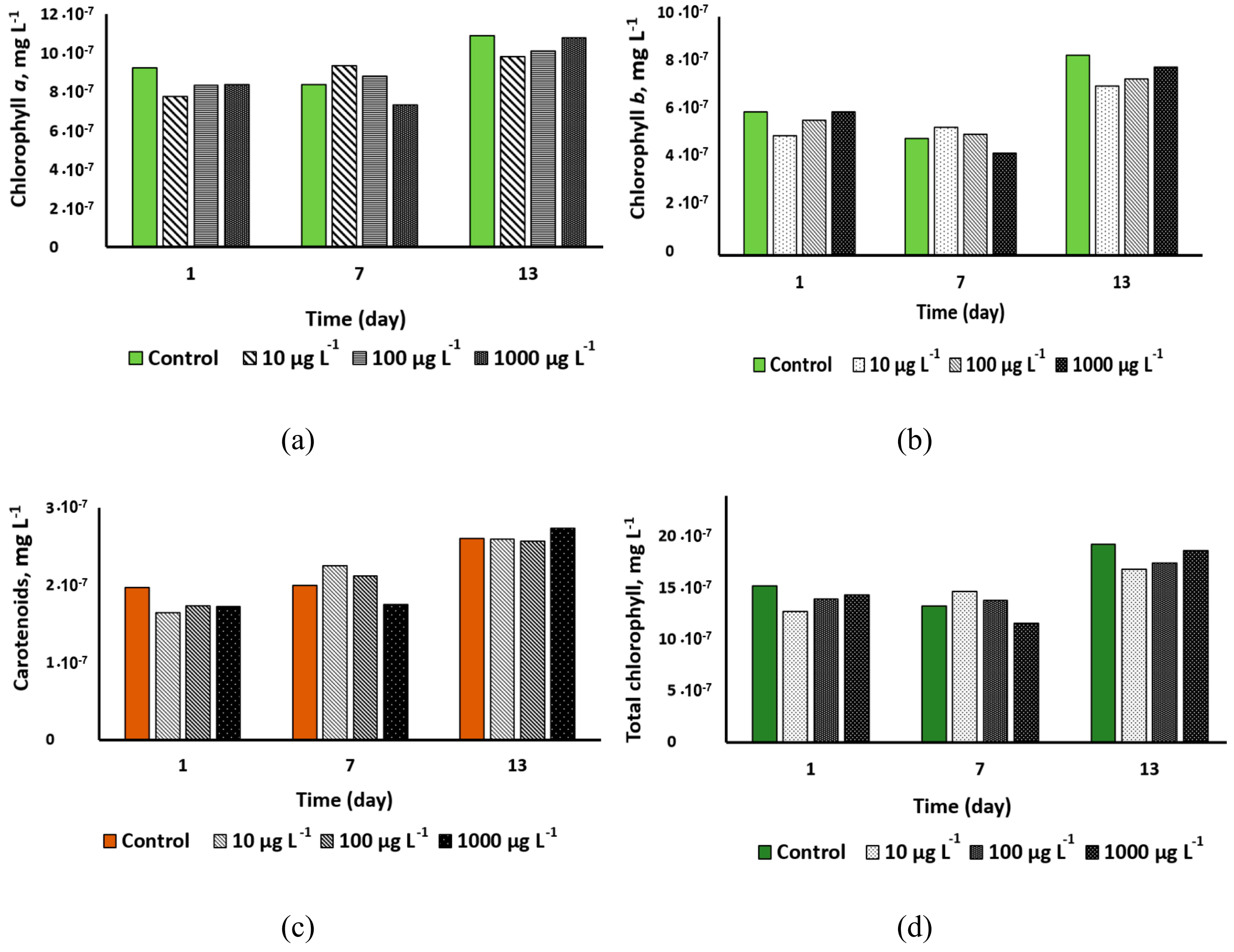

Figure S3. The dynamics of (a) chlorophyll a, (b) chlorophyll b; (c) total carotenoid, and (d) chlorophyll a + b content per cells of *Desmodesmus* sp. incubated in the presence of different concentrations of CuO-NP
